# The role of the AC component in human perception of AC–DC hybrid electric fields

**DOI:** 10.1038/s41598-022-07388-w

**Published:** 2022-03-01

**Authors:** Kathrin Jankowiak, Andrea Kaifie, Thomas Krampert, Thomas Kraus, Michael Kursawe

**Affiliations:** 1 Research Center for Bioelectromagnetic Interaction (femu), Institute for Occupational, Social and Environmental Medicine, Uniklinik RWTH Aachen University, Pauwelsstraße 30, 52074 Aachen, Germany; 2 Institute for Occupational, Social and Environmental Medicine, Uniklinik RWTH Aachen University, Aachen, Germany; 3 Institute for High Voltage Equipment and Grids, Digitalization and Power Economics, RWTH Aachen University, Aachen, Germany

**Keywords:** Human behaviour, Energy and behaviour, Electrical and electronic engineering, Environmental social sciences

## Abstract

Electric energy is essential to today’s society. To cope with global higher demand while minimizing land use, efficient high voltage direct current (HVDC) power lines are planned to be mounted on existing alternating current (AC) structures leading to electric fields (EFs) from both AC and DC transmission lines in hybrid configurations. Due to the close proximity to residential areas, the investigation of human hybrid EF perception and underlying mechanisms will be useful to project permitting. To specify the influence of the AC component on the whole-body detection thresholds of hybrid EFs and to explore the lower bound of human hybrid EF perception, 51 participants with an EF detection ability above average were exposed in a double-blind laboratory study. A psychophysical method based on the signal detection theory was used. Very low EF strength combinations, e.g. 1 kV/m AC combined with 1 kV/m DC, were reliably perceived by at least one participant. Detection thresholds were significantly lower with increased AC EF strengths, underlining the key role of the AC component in the human perception of hybrid EFs. Findings will contribute to the assessment of public reaction to the perception of EFs around hybrid overhead power lines and to their optimal designs.

## Introduction

In recent years, the technology for energy production and delivery has remarkably changed. New techniques of generating energy, especially from renewable power sources, combined with efficient high voltage direct current (HVDC) power lines were established to cope with higher long-distance transmission needs for electricity between generation and consumption areas. HVDC overhead power lines have already been built, especially in rural areas^1^. Now, to reduce costs and minimize the impact on the landscape and the need for new rights-of-way in built-up areas, HVDC lines are planned to be mounted on existing high voltage alternating current (HVAC) structures leading to AC–DC hybrid electric fields (hybrid EFs)^2^. Due to the close proximity to industrial and residential areas, the thresholds for human perception of hybrid EFs are of great interest. Moreover, no agency has proposed limit values for hybrid EF exposures.

Humans are able to reliably detect EFs^3,4^. So far, no specific receptors or signal transduction pathways have been clearly identified. However, hair receptors used for detection of vibration on hairy skin seem to be crucial for EF perception^5,6^. As Reilly suggested, the movement of charges along the hair shaft of a hydrated hair might lead to mutual repulsion between single hair follicles resulting in a perceptible vibration^4^. This process of AC EF perception is thought to be facilitated by an increased relative permittivity of body hair^7^. Interestingly, in a recent study, we found that higher skin moisture did not result in facilitated EF perception, at least when it was assessed at a single measurement point before starting the EF perception testing^8^. Moreover, body surface sensibility seems to be another important factor influencing EF perception. Kursawe et al. recently demonstrated a relationship between vibrotactile measures (31 Hz and 63 Hz) and the detection of AC EFs and hybrid EFs^8^. The authors explained the correlation with hybrid EFs by the kind of evoked sensation, i.e., tingling, itching, or vibration and stressed the importance of body surface sensibility in the interaction mechanism of EF perception^8^. Since large interindividual differences were found in human EF perception^3^, it is likely that differences in body surface sensibility or the length, structure, and relative permittivity of body hair all may influence individual EF perceptions.

To date, only a few experimental studies have focused on human perception of EFs. Local EF exposure showed rather heterogeneous detection thresholds of about 375 kV/m for DC EFs and 8–33 kV/m for AC EFs^5,6^. Under whole-body exposure and laboratory conditions, DC detection thresholds were estimated between 18.7 and 45.1 kV/m and decreased when ion currents were simultaneously generated^8,9^. Whole-body exposure only to AC EF produced detection thresholds between 14 and 25 kV/m^4,8^. The first investigations of human perception of hybrid EFs from overhead test lines were conducted by Clairmont et al.^10^. An unspecified number of participants were asked to judge the sensation level of EFs while standing under AC–DC overhead power lines. Averagely, a hybrid EF of 15 kV/m DC and 5 kV/m 60-Hz AC was rated as just perceptible^10^. Both increased DC EFs and increased AC EFs led to higher ratings of sensation levels in hybrid EFs. As the average perception rating of DC EFs alone was in the range of 20–25 kV/m, this nonblinded experimental field study provided the first hints that combined AC–DC EFs led to enhanced EF perception compared to either EF taken separately^10^. Increased sensation levels in hybrid EFs were also assessed when exposing four participants in a hybrid environment chamber^11^. In line with these findings, Kursawe et al. determined decreased detection thresholds of 6.76 kV/m DC combined with 4 kV/m 50 Hz AC under hybrid condition compared to detection thresholds of single DC EF or AC EF presentation (DC: 18.69 kV/m, AC: 14.16 kV/m) testing 203 participants in a well-controlled, double-blind laboratory study^8^. The results confirm differences between single EF and hybrid detection threshold estimates as described in our preliminary study^12^. This synergistic effect of the combined exposure of DC and AC EFs on human perception was supported by 40% of the participants being able to detect the lowest EF strength combination of 2 kV/m DC and 4 kV/m AC^8^. Within this first systematic examination of human hybrid EF perception, our group assessed facilitated detection performances in hybrid EFs when adjusting the DC component to higher EF strengths while keeping the AC EF constant at 4 kV/m.

In the current study, we aimed to specify the role of the AC component in the human perception of hybrid EFs. Fifty-one participants with a hybrid EF detection ability above average who participated in our previous study were exposed under highly controlled conditions to explore the human perception of low hybrid EFs. A secondary objective was to evaluate the influence of vibrotactile perception and skin moisture on EF perception, whereby skin moisture was repeatedly measured throughout the test day.

## Methods

### Participants

In this study, 51 healthy participants (20 men and 31 women) at the ages of 23 to 77 (mean 49.14, SD 17.52) were recruited from participants in our earlier study^8^. The requirement for participation was the ability to detect hybrid EF strength combinations of at least 4 kV/m AC and 8 kV/m DC, which was tested in the previous investigation. From 92 participants fulfilling this criterion, 51 responded to our invitation and were included in the study. Based on the medium effect size in previous research^8^, we calculated a test power of 99% to find an effect of EF strength. Exclusion criteria were electronic implants or indelible piercings, pregnancy, self-reported electrosensitivity, skin diseases, as well as psychiatric or neurological disorders. Signs of infection, as well as cutaneous or cardiovascular abnormalities, and medication or drug abuse were ruled out during the physical evaluation at the beginning of each test day. Risks and benefits of the study were thoroughly explained and all participants gave their written informed consent. The expense allowance was 100 Euro per participant. The ethics committee of the Medical Faculty of RWTH Aachen University approved the study (EK 065/20), which was conducted according to the Declaration of Helsinki without preregistration.

### Exposure laboratory

**Figure 1:**
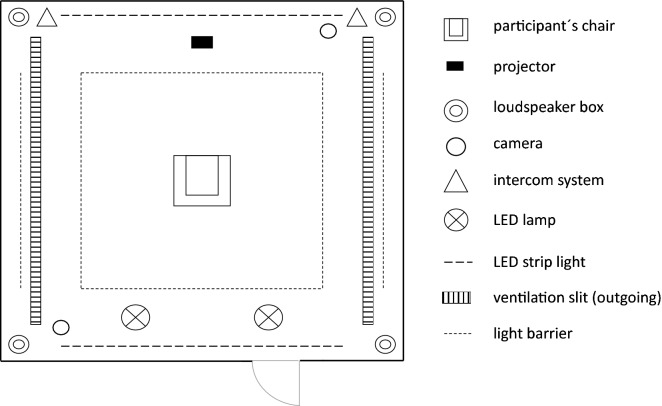
Schematic top view of the exposure laboratory. Adapted from Jankowiak et al.^12^.

EF perception tests were performed in a specially designed exposure laboratory, which was constructed at the University Hospital RWTH Aachen in cooperation with the Institute for High Voltage Equipment and Grids, Digitalization and Power Economics, RWTH Aachen University. A detailed description of the facility and the technical setup has been described in Jankowiak et al.^12^. In the middle of the 16 square meter exposure laboratory, a height-adjustable chair was installed (see Fig. 1). To avoid charge accumulations at the chair and the walls, laminated densified wood was used. Monitoring electrodes were placed on both ankles of the participants via low resistive connections to record induced body currents during exposure and to ensure a connection to the ground. To provide homogeneous test conditions, all participants were set to the same head height by adjusting the chair. Additionally, an intercom system transmitted all sounds from the exposure laboratory to the adjacent investigator room and allowed a direct communication with the participants. Background noise generated by the high-voltage system was masked by a 65.8 dB(A) incoherent white noise played in the exposure laboratory during experimental testing.

Four high-voltage electrodes in the ceiling of the exposure laboratory were used to generate EFs. In this study, various hybrid EFs with a 50 Hz AC component of up to 4 kV/m (in the current work, AC EF strengths always refer to rms values) and a DC component of up to 16 kV/m were generated by an aluminum electrode, which was energized by a 34 kV AC transformer combined with a 200 kV DC source. To generate a homogenous EF to the base plate on the floor, 14 grading electrodes were mounted on the walls one below the other connected via ohmic-capacitive grading units. Several calibration and check-up measurements before or after experimental testing ensured the correct generation of the required EF. A field-mill type probe was used to calibrate the applied DC EF, while the measured displacement current enabled the calculation of the AC EF. Both, AC EF and DC EF uniformity were similar to those measured in previous studies^12^. A proper functioning of the EF generating system was ensured by a laboratory technician.

For safety reasons, two cameras filmed the exposure laboratory permanently. Further security measures, such as light barriers around the participant’s chair and a seatbelt equipped with contact plugs at both ends, were implemented to ensure a sufficient safety distance towards the electrodes at any time. An interruption of both the light barriers and the contact plugs of the seatbelt led to a shut-down of the EF generating system within 180 ms. Furthermore, pressing one of the three emergency stops, placed next to the participant, the investigator, and the laboratory technician, immediately stopped the experimental testing.

### Measures of skin moisture and vibrotactile perception

To investigate the influence of individual factors on the perception of EFs, skin moisture and vibrotactile perception were recorded. Five measurements of skin moisture were conducted without parallel EF exposure throughout the test day to track possible variations and ensure a temporal proximity to EF exposure. Each measurement was performed three times on both insides of the forearms using a corneometer (CM 825; Courag + Khazaka electronic, Cologne, Germany). Skin moisture values were indicated dimensionless. Prior to the first EF exposure, vibrotactile perception was assessed with a perception meter (HVLab, Institute of Sound and Vibration Research, University of Southampton, Southampton, England). Vibrotactile perception was measured at 16, 40, 50, 63, and 200 Hz to cover a wide frequency range while focusing on the 50 Hz range. Participants were asked to press a button as long as they were perceiving a vibration in the right index finger. Pressing the button led to a decreasing amplitude of the vibration frequency, while not pressing the button was directly connected to an increasing amplitude until participants were again perceiving the vibration. Six reversals were used to calculate a mean value for each vibration frequency.

### Psychophysical method for EF detection

Analyses of psychophysical measures based on the signal detection theory (SDT), introduced by Green and Swets^13^, were used to estimate the participant’s ability to detect EFs and to calculate detection thresholds. The participant’s task was to discriminate a fixed number of exposure trials (EF present) from the same number of randomly intermixed sham trials (no EF present) by pressing a button on a response pad. Four different outcomes were possible depending on the participant’s response. An exposure trial could either be detected correctly (hit) or not perceived (miss). In line with this, sham trials could be classified as falsely perceived (false alarm) or correctly denied (correct rejection). To calculate the individual sensitivity (*d*′) towards a given EF, the relative proportion of hits and false alarms were z-transformed and subtracted [*d*′ = *z*(*hit*) − *z(false alarm)*]. Hit rates and false alarm rates of 0 and 1 were corrected by the log-linear procedure to enable a z-transformation^14,15^. A successful detection of a given EF was assumed when *d*′ values equal or greater than 1 were reached. While *d′* values between 1 and 2 reflect a successful detection performance, *d*′ values greater than 2 refer to a good sensitivity.

### Test procedure

Four different test conditions were designed with constant AC EF strengths of 1, 2, 3, or 4 kV/m (rms values) at a frequency of 50 Hz combined with varying DC EF strengths of 1, 2, 4, 8, or 16 kV/m leading to various total EF strengths (see Table 1). Each test condition was randomly carried out in two sessions with randomized trial sequence. Therefore, each participant performed eight sessions on a single test day. Within each session, 50% sham trials were randomly interspersed to ensure the correct usage of the SDT method. A double-blind experimental setup in which neither the participant nor the investigator was aware of the trial sequence within a given session was established under computer control.

**Table 1:** Four different test conditions were designed with constant 50 Hz AC EF strengths of 1, 2, 3, or 4 kV/m (rms values) and varying DC EF strengths of 1, 2, 4, 8, or 16 kV/m. Total EF strengths were calculated by $\sqrt {E_{{{\text{DC}}}}^{2} + E_{{{\text{AC}}}}^{2} }$. Each test condition consisted of two sessions with 20 exposure trials and 20 sham trials, respectively, whereby trial sequence differed in the second presentation.

	AC EF strength (kV/m)	DC EF strength (kV/m)	Total EF strength (kV/m)
Condition 1	1	1, 2, 4, 8, or 16	1.41, 2.24, 4.12, 8.06, or 16.03
Condition 2	2	1, 2, 4, 8, or 16	2.24, 2.83, 4.47, 8.25, or 16.12
Condition 3	3	1, 2, 4, 8, or 16	3.16, 3.61, 5.00, 8.54, or 16.28
Condition 4	4	1, 2, 4, 8, or 16	4.12, 4.47, 5.66, 8.94, or 16.49

One session lasted about 15 min and consisted of 40 trials. The length of each trial was fixed and independent of the applied EF strength. Within 3 s, the EF was increased from zero to the target EF strength and remained constant for 5 s. Then, a question was projected on the wall in front of the participants that asked if they perceived an electric field. During the 4 s response period, the desired EF strength was maintained. Participants answered by pressing a button on the response pad and could choose from four options: “Yes-certain”, “Yes-uncertain”, “No-uncertain”, and “No-certain”. Thereafter, the EF decreased within 7–9 s and the next trial started immediately. Sham trials followed the same timing, but no EF was applied. During the whole test day, the temperature and the relative humidity in the exposure laboratory were constant at 22 °C and 50%, respectively.

### Data processing and statistical analyses

Based on the *d*′ values derived from the SDT procedure, individual psychometric functions regarding the hybrid EF perception ability were calculated for each test condition. Referring to Kursawe et al.^8^, total EF strengths were calculated by $\sqrt {E_{{{\text{DC}}}}^{2} + E_{{{\text{AC}}}}^{2} }$ (see Table 1). Using linear interpolation, individual detection thresholds were determined, defined as the calculated EF strength where *d*′ = 1 was reached. Two constraints had to be considered: no detection threshold could be estimated when participants did not reach a *d*′ ≥ 1 in any EF strength or when participant’s data distribution over all EF strengths was inconsistent, e.g. a *d*′ ≥ 1 for 5 and 8.54 kV/m, but a *d*′ < 1 for 16.28 kV/m. Individual detection thresholds were averaged.

Skin moisture values, *d*′ values, and detection thresholds were analyzed in separate repeated measures analyses of variances (rm ANOVAs) using SPSS 25 (IBM, Armonk, NY). For rm ANOVAs on detection thresholds, the number of participants was reduced to n = 19, because data of 19 participants met preconditions for the calculation of detection thresholds in each test condition. The alpha level of *p* = 0.05 was accepted for significance. When sphericity was violated, Greenhouse–Geisser correction was applied and uncorrected degrees of freedom with corrected *F* and *p* values were indicated^16^. Partial eta-squared (η_p_^2^) values were used as a measure of effect size. Non-parametric correlations between the measures of individual factors and detection thresholds of each test condition were computed using Spearman’s ρ. The level of significance was corrected according to Bonferroni.

## Results

**Figure 2:**
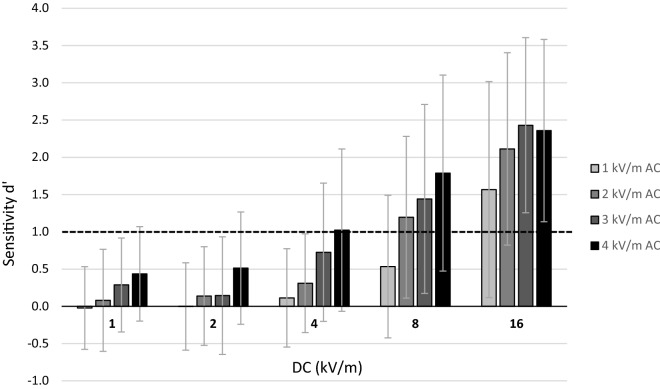
Influence of the AC component on DC sensitivities. Averaged sensitivities (*d′*) for AC EF strengths of 1, 2, 3, and 4 kV/m combined with DC EF strengths of 1, 2, 4, 8, and 16 kV/m. Bars reflect standard deviations. Black line represents *d′* of 1 indicating a successful detection.

Averaged *d*′ sensitivities of all EF strength combinations are illustrated in Fig. 2. The rm ANOVA with the factors *AC EF strength* (1, 2, 3, 4 kV/m) and *DC EF strength* (1, 2, 4, 8, 16 kV/m) showed a significant main effect of *AC EF strength* (*F*(3, 150) = 38.96, *p* < 0.001, η_p_^2^ = 0.44), as well as *DC EF strength* (*F*(4, 200) = 99.39, *p* < 0.001, η_p_^2^ = 0.67), and an interaction effect (*F*(12, 600) = 4.47, *p* < 0.001, η_p_^2^ = 0.08). Planned pairwise comparisons showed significant differences between all AC EF strengths (all *p’s* < 0.02). Except for the lowest DC EF level (1 and 2 kV/m), all DC EF strengths significantly differed as well (all *p’s* < 0.02). As sensitivities of *d*′ ≥ 1 reflect a successful detection, eight EF strength combinations were averagely perceived (4 kV/m AC with 4 kV/m DC; 2, 3, and 4 kV/m AC with 8 kV/m DC; all AC EF strengths with 16 kV/m DC). In general, both higher AC EF strengths and higher DC EF strengths increased *d*′ values. These effects correspond to the increased number of participants who successfully detected the respective EF strength combination with both higher AC EFs and higher DC EFs (see Table 2). Whereas only one participant showed a successful perception of the lowest EF strength combination (1 kV/m AC and 1 kV/m DC), 43 participants were able to detect the highest EF strength combination of 4 kV/m AC and 16 kV/m DC.

**Table 2:** Number of participants (out of n = 51) who successfully detected the respective EF strength combination (*d′* ≥ 1).

AC (kV/m)	DC (kV/m)				
	1	2	4	8	16
1	1	3	2	14	30
2	4	3	6	29	42
3	9	7	14	29	42
4	11	13	25	35	43

**Figure 3:**
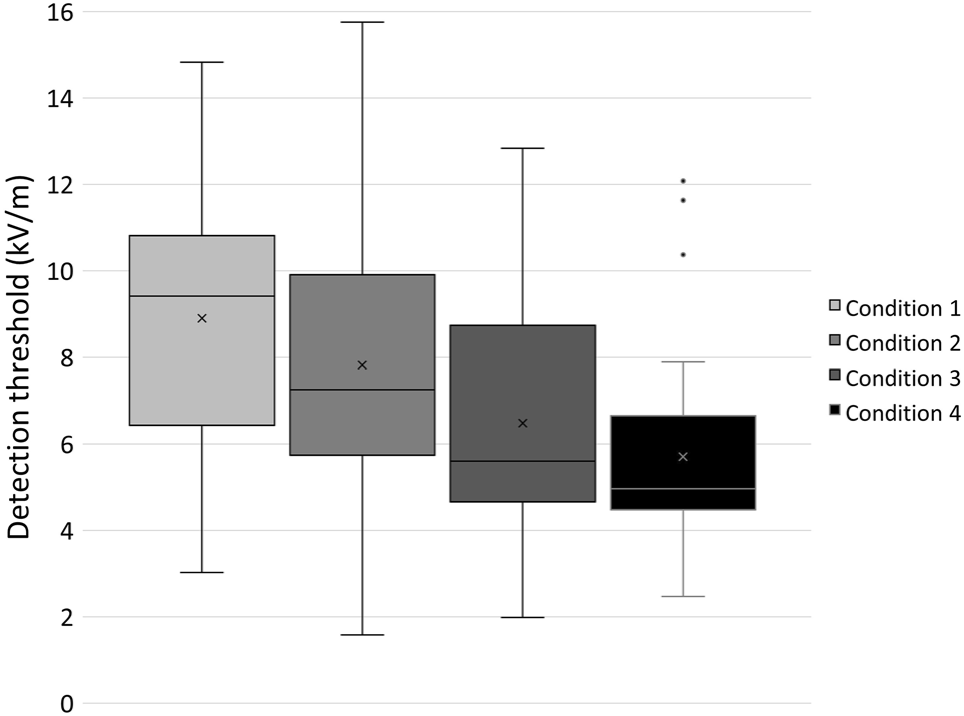
Boxplot diagrams with detection thresholds for all test conditions (DC EF strengths in all conditions were 1, 2, 4, 8, or 16 kV/m; AC component varied between conditions but was constant during one condition: 1 kV/m in Condition 1, 2 kV/m in Condition 2, 3 kV/m in Condition 3, and 4 kV/m in Condition 4). Detection thresholds based on total EF strengths ($\sqrt {{\text{E}}_{{{\text{DC}}}}^{2} + {\text{E}}_{{{\text{AC}}}}^{2} }$). Number of participants, on which the estimated detection thresholds are based, were 26, 38, 34, and 33 for Condition 1, 2, 3, and 4, respectively. Crosses indicate averaged detection thresholds. Median values are expressed by the horizontal bar within the boxes. Dots represent outliers. Whiskers indicate the minimum and the maximum value of each data set.

Based on the total EF strengths and the interpolation of individual sensitivity indices, reduced detection thresholds were observed with increased AC EF strengths (see Fig. 3). Averaged detection thresholds were 8.89 kV/m (SD 3.40), 7.82 kV/m (SD 3.17), 6.48 kV/m (SD 2.84), and 5.70 kV/m (SD 2.21) for test condition 1, 2, 3, and 4, respectively. The rm ANOVA on the four detection thresholds revealed a significant main effect (*F*(3, 54) = 20.55, *p* < 0.001, η_p_^2^ = 0.53) underlining the substantial influence of the AC component on the hybrid EF perception.

Two individual factors and their hypothesized association with EF perception were investigated (skin moisture and vibrotactile detection). Skin moisture was measured at five measurement points (MPs) throughout the test day and mean values of both arms were 55.38 (SD 15.42), 51.41 (SD 12.48), 51.97 (SD 13.63), 50.86 (SD 11.75), and 51.29 (SD 13.10) for MP1, MP2, MP3, MP4, and MP5, respectively. The rm ANOVA with the factor *Time* (MP1, MP2, MP3, MP4, MP5) revealed a significant main effect (*F*(4, 200) = 8.44, *p* = 0.001, η_p_^2^ = 0.93). Pairwise comparisons showed significant differences between MP1 and MP2, MP4, and MP5 (all *p’s* < 0.05) underlining significantly increased values at MP1. A conclusive link between skin moisture and detection thresholds was not found, though (all *p’s* > 0.68). Mean vibrotactile detection thresholds were 0.09 dB (SD 0.06), 0.24 dB (SD 0.14), 0.38 dB (SD 0.30), 0.47 dB (SD 0.40), and 0.43 dB (SD 0.48) for vibration frequencies of 16, 40, 50, 63, and 200 Hz, respectively. However, significant correlations between vibrotactical scores and detection thresholds could not be found (all *p’s* > 0.2).

## Discussion

The current study aimed to specify the role of the AC component in the human perception of hybrid EFs. Fifty-one participants with a hybrid EF detection ability above average were exposed under highly controlled conditions to explore the human perception of low hybrid EFs. Detection thresholds of hybrid EFs were significantly lower with increased AC EF strengths and even very low EF strength combinations of up to 1 kV/m AC EF combined with 1 kV/m DC EF were reliably perceived by at least one participant (*d*′ > 1). Correlations between vibrotactile detection thresholds and detection thresholds of hybrid EFs could not be replicated.

Both the sensitivity values and the number of participants who successfully detected the respective EF strength combination were higher with increased EF strengths, which also was reported in previous studies^8,9,12^. The factor *AC EF strength*, as well as *DC EF strength*, showed a significant main effect. Therefore, both EF types substantially influenced the hybrid EF perception, which is in line with previous findings^10,11^. The interaction effect of both factors was triggered by a constant performance of the participants at 2 and 3 kV/m AC with 2 kV/m DC, a lower performance at 4 kV/m AC with 16 kV/m DC compared to 3 kV/m AC with 16 kV/m DC, and a lower performance at 3 kV/m AC with 2 kV/m DC compared to 3 kV/m AC with 1 kV/m DC; bearing in mind that the two last differences were based on subthreshold sensitivity values.

This facilitating effect on the hybrid EF perception with increases in either the AC EF strength or the DC EF strength also was evident from the number of participants who were able to detect the respective EF strength combination (*d*′ ≥ 1) (see Table 2). Only one participant could successfully detect the lowest EF strength combination of 1 kV/m AC and 1 kV/m DC. Therefore, each EF strength combination was reliably detected by at least one participant underlining the human sensitivity to hybrid EFs. However, eight participants did not succeed in perceiving the highest EF strength combination of 4 kV/m AC and 16 kV/m DC. According to the inclusion criterion that hybrid EFs of at least 4 kV/m AC and 8 kV/m DC had to be reliably perceived in previous investigations, these participants did not meet expectations based on their performance in an earlier study^8^. This deviation might be explained by an altered EF strength presentation, which influenced the participant’s performance: In the current study, participants were exclusively exposed to low hybrid EFs, whereas in previous investigations, hybrid EF strength combinations of up to 4 kV/m AC combined with up to 24 kV/m DC were presented, as well as single AC and DC EFs with a maximum of 30 kV/m and 38 kV/m, respectively. Based on this altered context, especially the missing strong EFs, the clear discrimination between exposure and sham trials was probably not as easy as in the previous participation for some participants. Hence, other sensations might have been misinterpreted as EF perception resulting in *d*′ sensitivity indices below 1.

Differences between our detection thresholds and those assessed in other studies^8,10^ were mainly based on different calculation methods of detection thresholds and different test group populations. Kursawe et al.^8^ exposed an age- and sex-balanced group without prior experience in EF perception, while we only included participants who were evidently able to detect low hybrid EFs. Moreover, in the current study, detection thresholds were determined after calculating total EF strengths by $\sqrt {E_{{{\text{DC}}}}^{2} + E_{{{\text{AC}}}}^{2} }$, whereas Kursawe et al.^8^ and Clairmont et al.^10^ estimated single DC detection thresholds for constant AC EF strengths. For specifying the role of one EF component, such as the AC component, detection thresholds based on the total EF strength were more suitable. Increasing the constant AC EF strength in each test condition (1, 2, 3, and 4 kV/m) led to substantially decreased detection thresholds of hybrid EFs (see Fig. 3). Therefore, the AC component plays a key role in the human perception of hybrid EFs. In the current study, 30 participants showed a successful perception at a total EF strength of 16.03 kV/m including 1 kV/m AC, while 42 participants were able to reliably perceive a total EF of 16.12 kV/m including 2 kV/m AC (see Table 2). As hybrid EF perception is facilitated by a higher AC component, it should be considered that not only the total EF strength but the EF composition of AC and DC EF strengths is decisive for estimating hybrid detection thresholds, particularly in the light of mutual influence of both components^2^. Furthermore, the synergistic effect of AC and DC on EF perception, revealed in previous studies^8^, was verified by a significant main effect of the detection thresholds. Increased sensation levels in hybrid EFs combined with more vibrations of raised body hair were also observed previously^11^. Theoretical calculations indicated that the electric force on body hair is up to three times higher in hybrid EFs compared to single AC or DC EFs^17^, which might be a possible explanation for the increased hybrid EF perceptions.

In line with previous findings^8,9^, we revealed interindividual variances that might be partially based on physiological properties. Our data indicated that skin moisture values did not remain constant throughout the test day but were significantly higher at the beginning of the day, which could be based on initial excitement or previous activity. As in Kursawe et al.^8^, we did not find a correlation between skin moisture and EF perception. However, several studies have shown a conclusive link between hair moisture and EF perception^6,7,11^. In our study, measurements of skin moisture on glabrous skin may not reflect actual hair moisture content. In contrast to Kursawe et al.^8^, we did not find a correlation between vibrotactile thresholds and detection thresholds of hybrid EFs. This could be caused by the reduced dataset of participants, who met the preconditions for the calculation of detection thresholds (see Fig. 3). Since evoked sensations were shown to be mainly dependent on movement of hair and the detection of vibrations^5,6,11^, future investigations with more participants will help to identify receptors or signal transduction pathways of human EF perception.

### Limitations

When interpreting the detection thresholds of the current study, it is important to note that only participants with a very good ability to detect hybrid EFs (successful detection of 4 kV/m AC combined with 8 kV/m DC in previous investigations) were included in order to explore the lower bound of hybrid EF perception. Hence, the current detection thresholds are derived from a special group of participants and cannot be transferred to the entire population. In addition, participants were grounded during the exposure. Thus, detection thresholds reported in the current study may differ from detection thresholds when participants were partially or wholly isolated from ground as might apply to the public walking near or under HV transmission lines. Moreover, detection thresholds were calculated based on a reduced number of participants due to some preconditions. If participants did not successfully perceive even the highest EF strength within a given session, no individual detection threshold could be estimated. Furthermore, the rm ANOVA on detection thresholds was conducted based on a reduced number of participants because individual detection thresholds in all four test conditions could be estimated only for 19 participants.

## Conclusion

Within the current study, we showed that the AC component plays a key role in the human perception of hybrid EFs as increased AC EF strengths lowered detection thresholds of hybrid EFs. Therefore, not only the total EF strength but especially the AC component in a hybrid EF is decisive for estimating hybrid detection thresholds. Focusing on the lower bound of hybrid EF perception, it could be indicated that some people are even able to reliably detect very low hybrid EFs of up to 1 kV/m AC EF combined with 1 kV/m DC EF. This high sensibility, along with the synergistic effect of AC and DC, should be taken into consideration when assessing public reaction to the perception of EFs around hybrid overhead lines. In this context, the effect of environmental factors, such as relative humidity and ion currents, on the perception of low hybrid EFs could be investigated in future studies, as both factors were associated with lower detection thresholds^8^. Together with these findings, our data will help to prevent unwanted EF perception in nature and contribute to the construction of future hybrid overhead power lines.

## Data Availability

The datasets generated during the current study are available from the corresponding author on reasonable request.
